# Supplementary material for: Real-time tracking of metal nucleation via local perturbation of hydration layers
Source: Nat Commun. 2017 Oct 17;8:971. doi: 10.1038/s41467-017-01087-1 (PMC5645439; doi:10.1038/s41467-017-01087-1)
Supplement: Supplementary file 1 — Supplementary Information [file 41467_2017_1087_MOESM1_ESM.pdf]

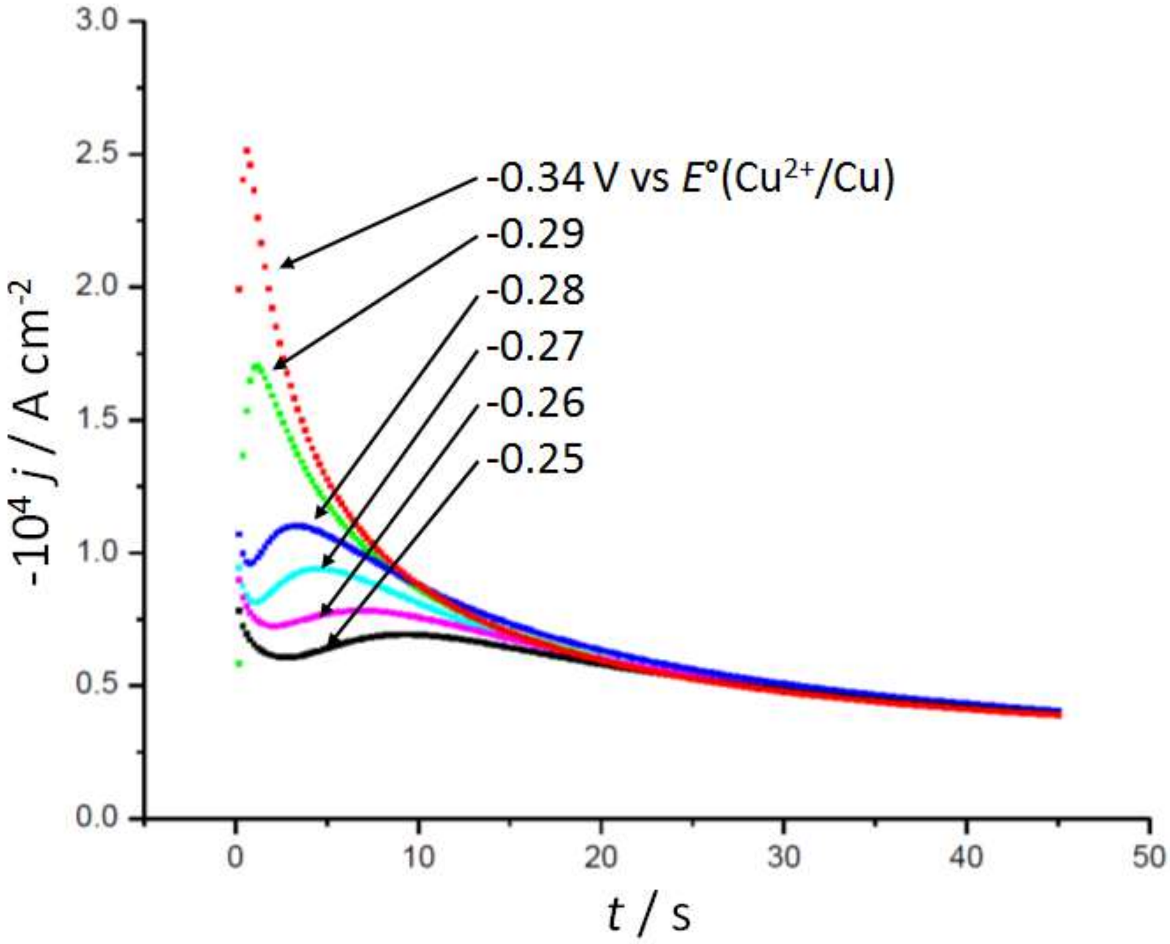

Supplementary Figure 1. Characteristic chronoamperometric transients at ITO electrodes in aqueous solution of $CuSO_4$ 1.0×10^{-3} mol×dm^{-3} at various overpotentials and room temperature. The electrolyte solution contained 50×10^{-3} mol×dm^{-3} Na_2SO_4 at pH 3.

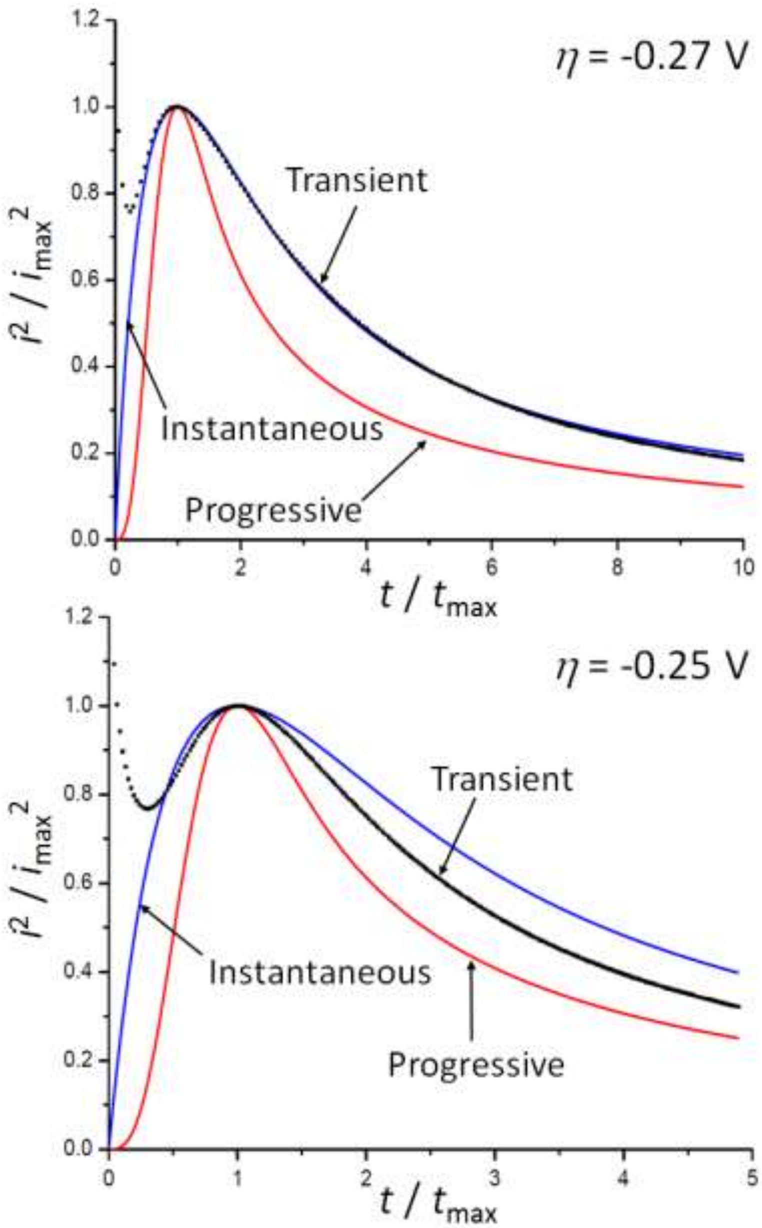

Supplementary Figure 2. Dimensionless plot of the current (*i*) normalised by the maximum current (i_{max}) as a function of time (*t*) at two overpotential. The experimental conditions are described in the caption of **Supplementary Figure 1**. The *instantaneous* and *progressive* nucleation limits as predicted by the Scharifker-Hills model[1] are indicated.

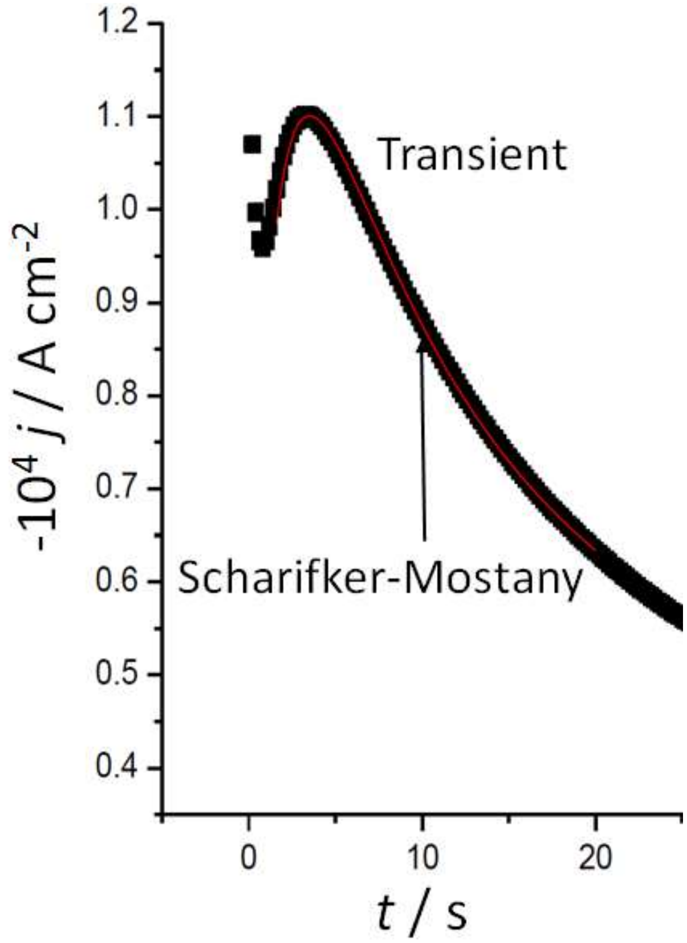

Supplementary Figure 3. Chronoamperometric transient recorded at -0.27 V fitted to the Scharifker-Mostany model.[2] Concentration of $CuSO_4$ is 1.0×10^{-3} mol$\times$dm^{-3}. Nuclei number density and nucleation rate of 5.3×10^{6} cm^{-2} and 15 s^{-1}, respectively, were estimated from the fit.

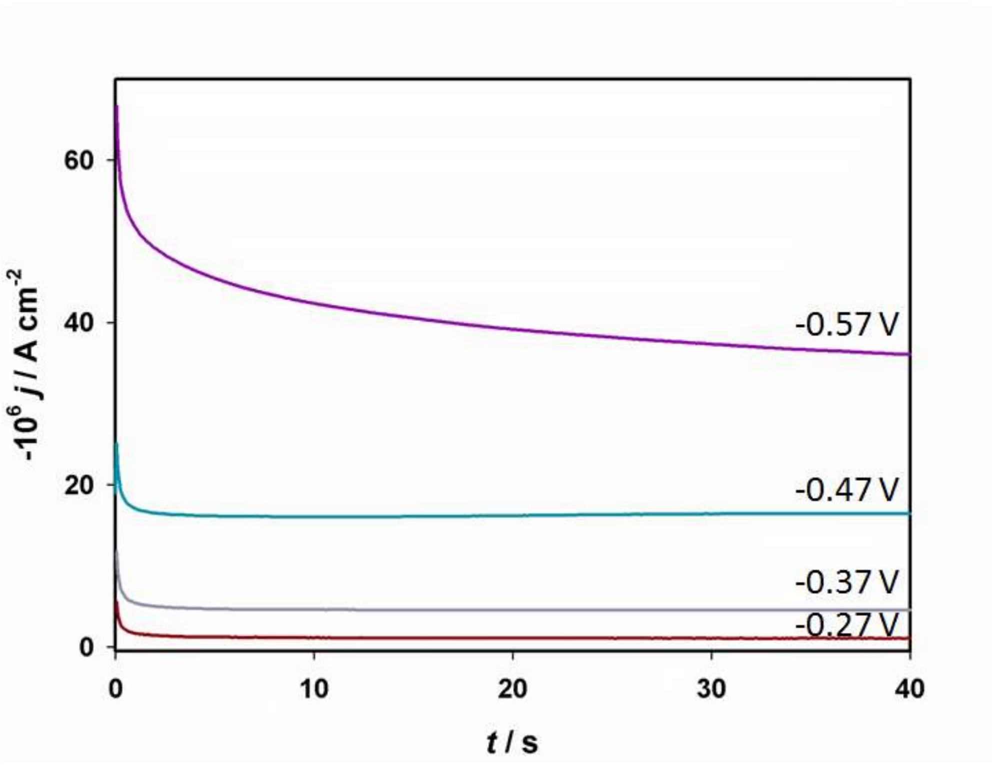

Supplementary Figure 4. Chronoamperometric transients recorded at various overpotentials on ITO electrodes at a $CuSO_4$ concentration of 1.0×10^{-4} mol×dm^{-3}. Other experimental conditions are described in the caption of **Supplementary Figure 1**. The lack of a well-defined current peak and diffusion limiting current suggest a convolution of the dynamics of electron transfer and nucleation.

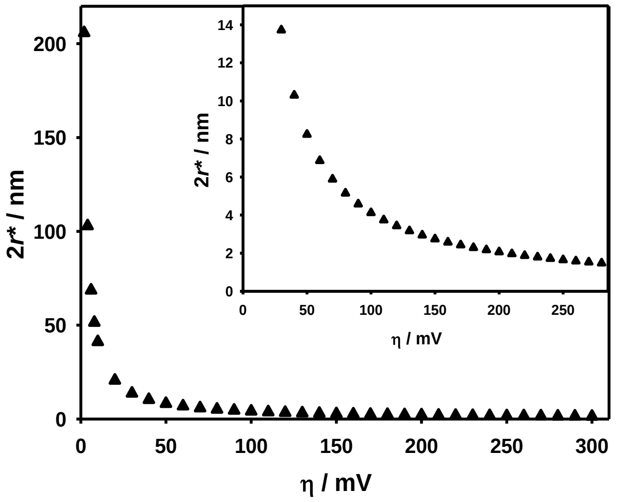

Supplementary Figure 5. Critical nucleus diameter ($2r^*$) as a function of the overpotential (η), estimated from **equation 1**. In **equation (1)** σ represents the surface tension, M is the atomic weight (M) and ρ is the density.[3] This simple relation predicts stable Cu nuclei of 1.5 nm diameter can be formed at overpotentials of -0.27 V. Our experimental observations based on HS-LMFM images revealed stable nuclei of the order of 6-7 nm. Considering that no tip-shape deconvolution routines were applied to these images, our experimental values are close to the thermodynamic limit.

$$r^* = 2\sigma M / \rho z F |\eta| \tag{1}$$

Supplementary References

1 Scharifker, B. & Hills, G. Theoretical and Experimental Studies of Multiple Nucleation. *Electrochim Acta* **28**, 879-889 (1983).

2 Scharifker, B. R. & Mostany, J. 3-Dimensional Nucleation with Diffusion Controlled Growth.1. Number Density of Active-Sites and Nucleation Rates per Site. *J Electroanal Chem* **177**, 13-23 (1984).

3 Scharifker, B. R. & Mostany, J. in *Encyclopedia of Electrochemistry: Interfacial Kinetics and Mass Transport* Vol. 2 (eds A.J. Bard, M. Stratmann, & E.J. Calvo) 512-540 (Wiley, 2003).
