# Supplementary material for: Real-time tracking of metal nucleation via local perturbation of hydration layers
Source: Nat Commun. 2017 Oct 17;8:971. doi: 10.1038/s41467-017-01087-1 (PMC5645439; doi:10.1038/s41467-017-01087-1)
Supplement: Supplementary file 3 — Description of Additional Supplementary Files [file 41467_2017_1087_MOESM3_ESM.pdf]

Description of Additional Supplementary Files

File Name: Supplementary Movie 1
Description: Initials stages of Cu nucleation and growth at 58 x 58 nm ITO surface during 49 s, at a constant overpotential of -0.27 V."

File Name: Supplementary Movie 2
Description: Growth on a pre-formed stable Cu nucleus on the ITO surface over a period of 57 s at an overpotential of -0.27 V. The final frame shows a superimposed 3D image of the particular growth (as in Figure 5c)."
